# CRISPR/Cas Genome Editing and Its Applications in Cereal Crop Improvement

**DOI:** 10.1002/pei3.70133

**Published:** 2026-02-24

**Authors:** Sirisha Kaniganti, Himanshu Saini, A. K. Chaitanya, Niranjan Hegde, Priya Shah, Nakul D. Magar, Ramesh Rijal, Jeevan Jyoti Kaushik, Deepak Nanda, Sharad Sachan, Anand Kumar, Roopali Bhoite, Harsha Rayudu Jamedar

**Affiliations:** ^1^ International Institute of Tropical Agriculture Nairobi Kenya; ^2^ School of Applied Natural Science Adama Science and Technology University Adama Ethiopia; ^3^ School of Agriculture Dev Bhoomi Uttarakhand University Dehradun Uttarakhand India; ^4^ Centre for Crop Health University of Southern Queensland Toowoomba Queensland Australia; ^5^ McGill University Montreal Quebec Canada; ^6^ International Crops Research Institute for the Semi‐Arid Tropics Patancheru Telangana India; ^7^ ICAR‐Indian Institute of Rice Research Hyderabad India; ^8^ Nepal Agricultural Research Council (NARC) Kathmandu Nepal; ^9^ School of Basic & Applied Sciences Shri Guru Ram Rai University Dehradun Uttarakhand India; ^10^ Tula's Institute of Pharmacy Dehradun Uttarakhand India; ^11^ Department of Agricultural Economics and Extension, School of Agriculture Lovely Professional University Punjab India; ^12^ Faculty of Agricultural Sciences GLA University Mathura Uttar Pradesh India; ^13^ Department of Primary Industries and Regional Development South Perth Western Australia Australia; ^14^ ICAR‐Indian Institute of Oilseeds Research Hyderabad India

**Keywords:** cereal crops, CRISPR/Cas genome editing, grain quality, stress tolerance, yield improvement

## Abstract

CRISPR/Cas‐based genome editing has emerged as a transformative tool for precise genetic improvement of cereal crops. Recent advances in CRISPR technologies, including Cas9, Cas12, Cas13, base editing, and prime editing, have enabled targeted modification of genes and regulatory elements controlling yield, stress tolerance, and grain nutritional quality in major cereals such as rice, wheat, maize, and barley. This review summarizes current progress in CRISPR‐mediated genome editing systems, delivery strategies, and representative applications in cereal crop improvement. Emphasis is placed on how genome editing reprograms enzymatic activities and biological pathways underlying complex agronomic traits rather than acting through single‐gene effects. The review also discusses challenges related to trait complexity, regulatory considerations, and prospects for translating genome‐edited cereal crops from laboratory research to field‐level application. Collectively, this review highlights the potential of CRISPR/Cas genome editing as a powerful approach for developing high‐yielding, resilient, and nutritionally improved cereal crops.

## 1 Introduction

Cereals have been a staple food in our diet since the beginning of agricultural practice, owing to their significant health benefits, nutritional content, and production. Cereals are high in complex carbohydrates, which supply us with a lot of energy. Cereals are a good source of protein, lipids, fats, vitamins, minerals, and fiber. Cereal crops are a fundamental source of dietary energy for the global population and underpin food and nutritional security worldwide. Staple cereals such as rice, wheat, and maize collectively provide a substantial proportion of daily caloric intake, particularly in low and middle‐income countries, and remain indispensable for meeting global food demand. Recent global assessments highlight that these three cereals alone dominate human calorie consumption and agricultural production systems, making their sustained productivity critical under conditions of population growth and climate change (FAO et al. 2024; Lemenkova 2025). Ensuring a stable supply of rice, wheat, and maize while simultaneously improving nutritional quality and resilience to climate‐induced stresses presents a major challenge that necessitates the adoption of innovative and precision‐driven crop breeding technologies.

**FIGURE 1:**
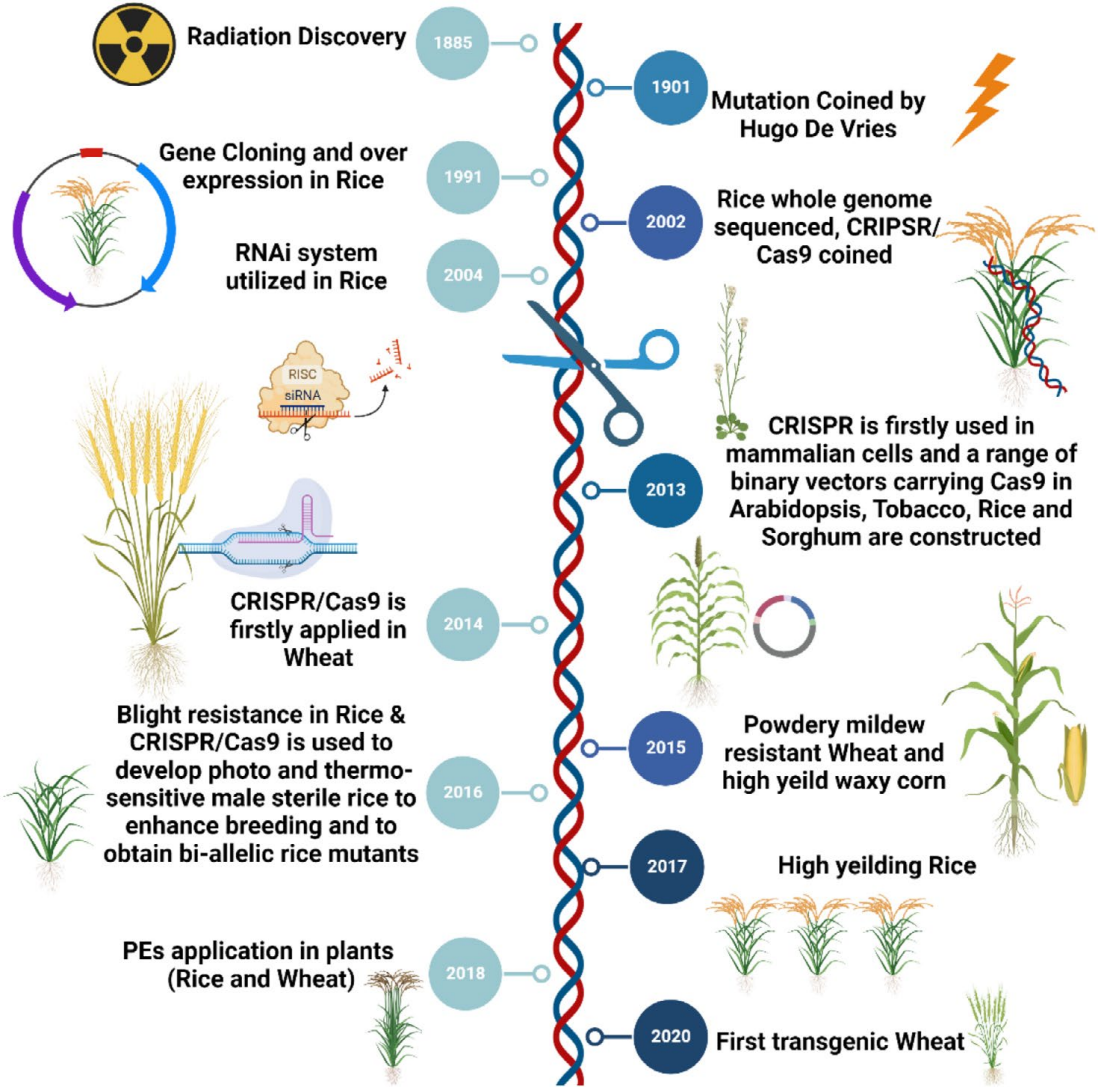
Overview of CRISPR/Cas‐based genome editing strategies and their applications in major cereal crops, including rice, wheat, maize, and barley, highlighting trait improvement targets such as yield, stress tolerance, and grain quality. (Figure created by the authors using BioRender.com).

Sequence‐specific nuclease‐based technology, CRISPR/Cas9 (Clustered Regularly Interspaced Short Palindromic Repeats/CRISPR‐associated protein 9) is a genome editing system derived from the adaptive immune mechanism of bacteria and archaea. The system comprises a Cas9 endonuclease guided by a programmable single‐guide RNA (sgRNA) that directs sequence‐specific recognition and cleavage of target DNA, enabling precise and efficient genetic modifications in eukaryotic genomes. Given their relevance, genome editing techniques have been widely used to improve cereal crops, allowing for the development of new varieties with improved yield and quality: Figure 1. The ability to make controlled alterations to the genome using certain nucleases is referred to as genome editing which causes recombination by inducing double‐strand breaks (DSBs) is a breakthrough in plants. These techniques have enabled site‐directed mutagenesis and gene replacement, which will contribute to crop improvement and advancement in functional genomics research. Over the past 3 years, CRISPR/Cas‐based genome editing in cereal crops has shifted decisively from early proof‐of‐concept mutagenesis toward pathway‐informed precision breeding. Recent studies emphasize multiplex genome editing, regulatory‐region engineering, and high‐precision base and prime editing to reprogram hormone signaling, carbon and nitrogen metabolism, stress perception, and immune responses in cereals, demonstrating the transition of CRISPR/Cas systems from experimental tools to field‐relevant breeding technologies (Zaidi et al. 2020; Kang et al. 2025; Vats et al. 2024).

Genome editing techniques, when combined with other current breeding strategies, availability of open‐source data of genes, and single nucleotide polymorphisms (SNPs) involved in important cereal traits can result in long‐term yield gains. It is now feasible to quantify gene expression and, using new approaches, to create knockout plants for various genes, allowing researchers to better understand the roles and functions of genes as well as their consequences in changing environments (Li et al. 2017).

The advent of CRISPR/Cas‐based genome editing has enabled efficient, programmable, and highly specific modification of endogenous genes in cereal crops (Zhang, Li, et al. 2018). Early demonstrations of CRISPR/Cas9‐mediated targeted mutagenesis in rice, maize, and wheat established its superiority over previous gene targeting approaches by enabling precise knockout and regulatory‐region editing at substantially higher efficiencies. More recent developments, including CRISPR‐mediated base editing and prime editing, have further expanded functional genomics capabilities by allowing predictable nucleotide substitutions and precise sequence insertions without inducing double‐strand breaks. In parallel, CRISPR‐based epigenome editing platforms have enabled targeted modulation of DNA methylation and chromatin states, providing direct and mechanistically relevant tools for studying gene regulation and complex trait control in cereals.

The genome editing technology has also been used to knock out disease susceptible genes and hence can generate resistant cultivars. All of these approaches have been used to change specific genes/loci in many cereal crops since ZFNs, TALENs, and CRISPR/Cas have progressed. Various investigations targeting different genes in maize, rice, wheat, and barley were carried out and enhanced GT trials. Because of its acceptance, cost‐effectiveness, reduced time required, and better and specific targeting, the CRISPR/Cas9 technology has been successfully used in important crops, particularly grain crops. The significant advancements in genome editing tools are projected to eliminate the defects and concerns associated with transgenic technology and supplant the transgenic development technique, at least for the time being. Earlier programmable nucleases such as ZFNs and TALENs laid the groundwork for targeted genome modification in plants, but the advent of CRISPR/Cas systems dramatically improved scalability, efficiency, and applicability in cereal crop improvement.

The current chapter focuses on recent advances in genome editing techniques that could lead to the development of new cereal crop types with increased yield, stress tolerance, and nutritional quality. We provide a summary of current complete breakthroughs in the CRISPR/Cas9 system, interesting applications, and crop enhancements in this chapter. The various genome editing technologies are discussed, with a focus on cereal model species applications. Recent advances in genome editing technology have displaced the limitations of traditional breeding tactics, ushering in a new era of crop improvement.

While early applications of genome editing focused on validating gene function, recent advances in CRISPR/Cas9 technology have shifted toward improving complex agronomic traits in cereal crops. Yield, stress tolerance, and grain quality are polygenic traits regulated by interconnected genetic and physiological networks. Consequently, effective crop improvement requires coordinated modification of multiple genes, regulatory elements, and signaling pathways rather than single‐gene interventions. This review therefore focuses on CRISPR‐mediated trait improvement in cereals, emphasizing yield stability, abiotic and biotic stress amelioration, and nutritional enhancement, rather than detailed descriptions of genome editing tools.

Grain yield is a systems‐level trait resulting from coordinated regulation of carbon assimilation, hormone homeostasis, nitrogen metabolism, and stress‐responsive pathways. Consequently, alteration of a single gene does not directly improve yield unless it induces measurable changes in host metabolism and enzymatic fluxes. Genome editing therefore contributes to yield improvement only when targeted gene modifications reprogram key metabolic steps or regulatory nodes within these interconnected pathways. This review adopts this metabolic perspective to analyze how specific CRISPR‐induced gene edits influence defined biochemical processes that collectively determine yield in cereal crops.

Given the inherent complexity of agronomic traits and the limitations of single‐gene explanations, this review deliberately focuses on a limited number of traits in cereal crops that are well characterized at the biochemical and physiological levels. Specifically, grain yield architecture, drought tolerance, grain quality (starch metabolism), and biotic stress resistance are discussed in depth to illustrate how CRISPR/Cas‐mediated genome editing reprograms enzymatic activities and metabolic pathways underlying these traits. Other reported applications are summarized more concisely to maintain analytical depth and coherence.

Although genome editing has also been explored in other crop groups, this review specifically focuses on cereal crops to provide a coherent and in‐depth analysis of CRISPR/Cas applications in these globally important species.

## 2 Harnessing Plant DNA Repair Pathways for Precision Genome Editing

**FIGURE 2:**
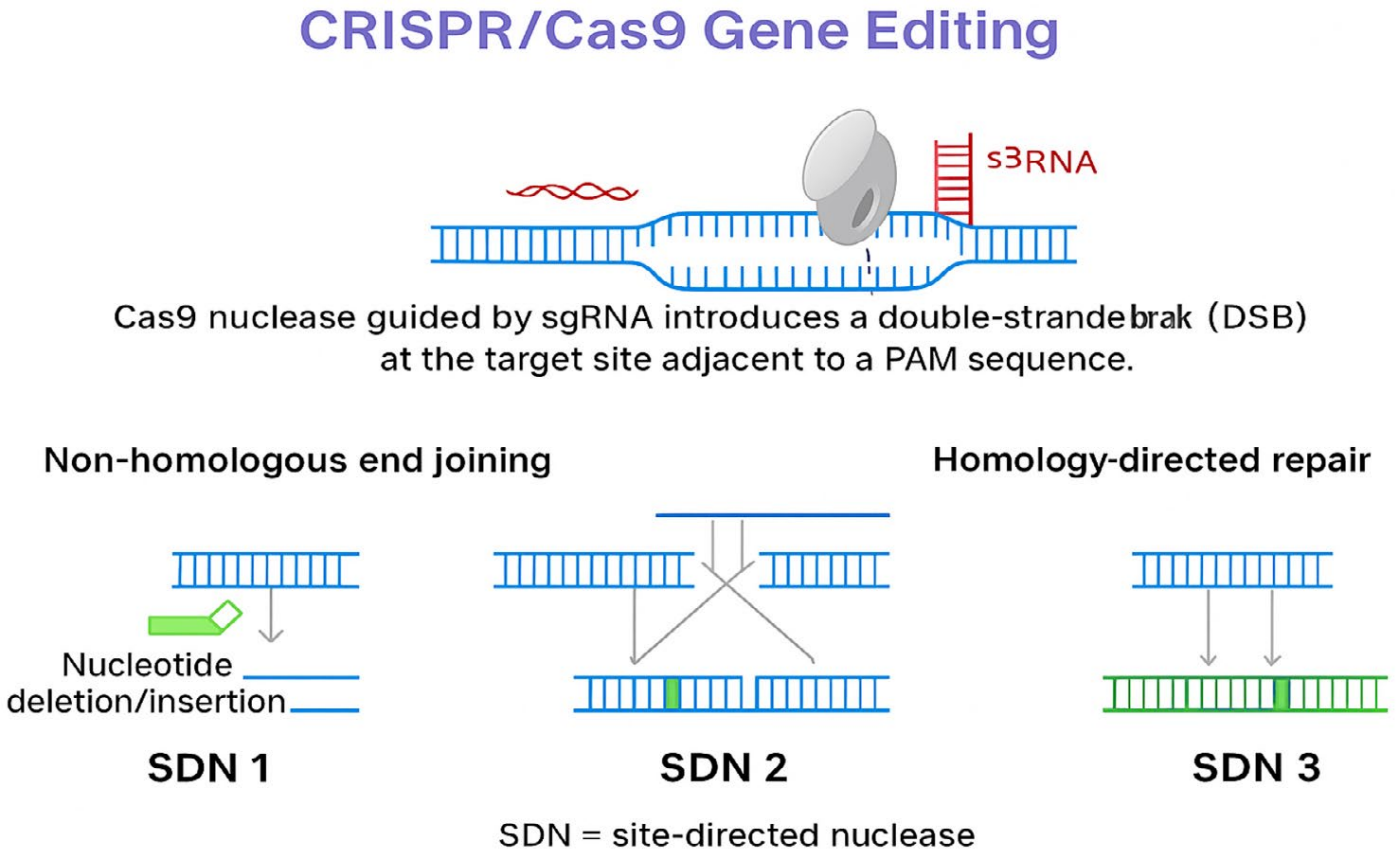
CRISPR/Cas9 mediated genome editing mechanism and the subsequent DNA repair pathways. Cas9 nuclease guided by sgRNA introduces a double‐stranded break (DSB) at the target site adjacent to a PAM sequence. Repair occurs via (i) non‐homologous end joining (NHEJ), generating insertions/deletions (SDN1), or (ii) homology‐directed repair (HDR) using donor DNA templates. HDR enables either precise small sequence changes (SDN2) or larger gene insertions/replacements (SDN3). SDN, site‐directed nuclease (Figure created by the authors using BioRender.com).

The CRISPR/Cas protein endonuclease is derived from a variety of bacterial species, including *Staphylococcus aureus, Streptococcus thermophilus*, and *Francisella novocida*, with *Streptococcus pyogenes* being the predominant source. The SpCRISPR/Cas9 system primarily identifies protospacer adjacent motif (PAM) (5′‐NGG‐3′) and cleaves target DNA just three to four bases upstream of PAM sequences to generate blunt‐end double‐strand breaks (DSBs). Following cleavage, the repair of the DSB is carried out by endogenous cellular mechanisms, leading to different editing outcomes (Figure 2). The non‐homologous end joining (NHEJ) pathway often introduces small insertions or deletions (InDels), resulting in gene disruption or knockout, commonly referred to as Site‐Directed Nuclease 1 (SDN1). Alternatively, the homology‐directed repair (HDR) pathway utilizes an exogenous donor DNA template to generate precise modifications. Depending on the design of the donor template, HDR can produce small, targeted nucleotide substitutions (SDN2) or facilitate the insertion or replacement of larger DNA fragments (SDN3). Together, these repair pathways form the basis of CRISPR/Cas9 applications in functional genomics and crop improvement, enabling both loss‐of‐function mutations and precise trait modifications in cereal crops. Throughout this review, CRISPR/Cas‐based applications are discussed in the context of this SDN framework to distinguish gene knockouts (SDN‐1), precise nucleotide substitutions (SDN‐2), and targeted sequence insertions or replacements (SDN‐3), particularly with respect to trait modification strategies and regulatory implications.

### 2.1 CRISPR/Cas9

The CRISPR/Cas9 system was developed to overcome the limitations of ZFNs and TALENs and has carved out its own niche in molecular biology. The CRISPR/Cas9 system, which consists of an array of CRISPR repeat‐spacers and Cas proteins that provide phage resistance to bacteria, has been cleverly adapted for genome editing applications. CRISPR/Cas systems are broadly classified into Class I and Class II based on the composition of their effector complexes. Class I systems employ multi‐subunit protein complexes to mediate target recognition and cleavage, whereas Class II CRISPR systems utilize a single, multidomain Cas effector protein (such as Cas9, Cas12, or Cas13) that associates with a guide RNA complex—either a crRNA‐tracrRNA duplex or an engineered single‐guide RNA (sgRNA)—to achieve target recognition and cleavage.

Inducing sequence alterations, such as knock‐ins, knock‐outs, exchange, genetic screening, imaging, etc., has been demonstrated to be a more versatile application of the class II system over time. Within class II, the CRISPR/Cas9 system has been utilized in a variety of plant species, ranging from model to crop plants, for the effective introduction of traits such as disease resistance (Oliva et al. 2019), nutrition, and climate‐resilience. The CRISPR/Cas9 framework includes Pol III promoters such as U3 or U6, a non‐repetitive 20‐bp region (guide RNA), and the Cas9 nuclease. The Cas9 proteins, which are the single protein effectors of type II CRISPRs, identify a PAM sequence and cleave three to four bases upstream of PAM, resulting in double‐stranded breaks (DSBs) and mutations and deletions at predetermined sites in the target genomes. Table 1 lists the genes edited in wheat crop using CRISPR technology, highlighting their roles in trait improvement.

**TABLE 1:** Genes edited in wheat using CRISPR technology.

Target gene(s)	Trait improved	Promoter/sgRNA–Cas	No. of gRNAs	Selectable marker	Delivery method	Genotype	Explant	Editing frequency (%)	Mutation type	Inheritance	References
*TaGW2, TaGASR7, TaDEP1, TaNAC2*	Grain size and yield improvement	Maize ubiquitin/TaU6	Multiple	—	Particle bombardment	Bobwhite, Kenong199	Protoplasts	3–32	INDEL	Heritable	Wang et al. (2018)
*TaASN2*	Reduced free asparagine (low acrylamide risk)	Maize ubiquitin	4	Bar	Particle bombardment	Cadenza	Immature embryos	—	INDEL	Heritable	Raffan et al. (2021)
*TaDREB2, TaERF3*	Enhanced drought tolerance	U6/ubiquitin	Multiple	Bar	*Agrobacterium*	Fielder	Immature embryos	12–25	INDEL	Stable inheritance	Bohra et al. (2024)
*TaARE1*	Improved nitrogen use efficiency and yield stability	U6/ubiquitin	2	Hygromycin	*Agrobacterium*	Fielder	Immature embryos	~20	INDEL	Heritable	Mahajan (2025)
*TaCKX6‐D1*	Increased grain number and yield	TaU6/ubiquitin	2	Bar	*Agrobacterium*	Fielder	Immature embryos	15–30	INDEL	Stable inheritance	Qian et al. (2025)
*TaGW7*	Grain width and thousand‐grain weight	TaU6/ubiquitin	1	Hygromycin	*Agrobacterium*	Jimai22	Immature embryos	28–35	INDEL	Homozygous in T1	Feng (2025)
*TaNRT2.1*	Improved nitrogen uptake efficiency	U6/ubiquitin	2	Bar	*Agrobacterium*	Fielder	Immature embryos	~18	INDEL	Heritable	Zhang et al. (2024)
*TaZIP4‐B2*	Meiotic stability and yield resilience	TaU6	1	Hygromycin	*Agrobacterium*	Chinese Spring	Immature embryos	~25	INDEL	Stable inheritance	Sourdille et al. (2025)

Engineered Cas9 variants with relaxed PAM requirements have expanded editable genomic space in cereals, facilitating allele and regulatory‐region engineering for complex trait modification. Table 2 summarizes recent CRISPR/Cas variants and their applications in major crop species.

**TABLE 2:** Recent CRISPR/Cas variants and their applications in major crop species.

CRISPR variant	Crop example (gene/allele edited)	Delivery method (how edits were introduced)	SDN category	References
SpCas9 (wild‐type) (DSB → NHEJ/HDR)	Rice: *OsSWEET14* promoter/coding disruption generating bacterial blight resistance	*Agrobacterium*‐mediated T‐DNA transformation with segregation to obtain edited lines	SDN‐1 (gene knockout via NHEJ)	Zeng et al. (2020)
SpCas9 (general knockouts/trait discovery)	Various crops: knockout of susceptibility or negative‐regulator genes (e.g., *OsERF922* in rice)	*Agrobacterium* transformation or particle bombardment; species‐dependent regeneration	SDN‐1	Gan and Ling (2022)
Engineered SpCas9 variants (SpCas9‐NG, xCas9, SpG, SpRY)	Arabidopsis/rice: allele engineering at expanded PAM sites	*Agrobacterium* T‐DNA or protoplast‐based plasmid delivery	SDN‐1 or SDN‐2*	Nishimasu et al. (2018)
Cas12a (Lb/As/Mb2/Mb3) (T‐rich PAM; multiplexing)	Rice: multiplexed gene knockouts with high biallelic efficiency	*Agrobacterium* T‐DNA expressing Cas12a+crRNA arrays	SDN‐1	Zhang, Zhang, et al. (2021) Zhang, Wang, and Li (2021), Zhang, Malzahn, et al. (2021)
Temperature‐tolerant/PAM‐relaxed Cas12a variants	Rice: increased genome coverage at AT‐rich loci	*Agrobacterium* delivery; stable and transient assays	SDN‐1	Zhang, Zhang, et al. (2021) Zhang, Wang, and Li (2021), Zhang, Malzahn, et al. (2021)
Cytosine base editors (CBE; C → T)	Rice: *OsALS* codon substitutions conferring herbicide resistance	*Agrobacterium* T‐DNA expressing nCas9‐deaminase fusions; transgene‐free segregation	SDN‐2	Zhang et al. (2020)
Adenine base editors (ABE; A → G)	Rice/tomato: precise nucleotide substitutions at trait loci	*Agrobacterium* or protoplast‐based transient expression	SDN‐2	Fan et al. (2024)
Transversion base editors (C → G; CGBE)	Rice/tomato: proof‐of‐concept transversion edits	Plasmid delivery via *Agrobacterium* or protoplasts	SDN‐2	Zong et al. (2022)
Dual/multiplex base editors (CBE+ABE)	Rice/tomato: simultaneous multi‐site base substitutions	Single T‐DNA constructs or multiplexed guide architectures	SDN‐2	Zong et al. (2018)
Prime editing (PE2/PE3)	Rice/wheat: precise base substitutions and small indels (e.g., *OsALS*)	*Agrobacterium* T‐DNA delivering nCas9‐RT+pegRNAs	SDN‐2	Li, Lin, and Zhang (2022)
CRISPR‐HDR (DSB+donor template)	Rice: targeted allele replacement/knock‐in cassettes	Particle bombardment or *Agrobacterium* delivering donor templates	SDN‐3	Zhou et al. (2025)
dCas9/CRISPRi/CRISPRa/epigenetic editors	Tomato/Arabidopsis: transcriptional regulation without DNA sequence change	*Agrobacterium*‐mediated transient or stable expression	Not SDN‐classified (no DNA modification)	Moradpour and Abdulah (2019)

### 2.2 Cas12

Cas12a (formerly designated as Cpf1) is a single RNA‐guided endonuclease system that belongs to the class II Cas system and type V‐A subgroup. Cas12a endonuclease does not require any trans‐activating CRISPR‐RNA (tracrRNA) and makes use of its own guideRNAs, resulting in enhanced editing capability.

Cas9 identifies the NGG PAM and generates blunt‐end DNA breaks upstream of the PAM site, whereas the Cas12a system recognizes the TTTV PAM and generates staggered DNA breaks downstream of the PAM site. Cas12a is superior to Cas9 in its ability to modify AT‐rich areas, and it cleaves the target DNA 18–23 nt downstream of the PAM without affecting the PAM region. During NHEJ repair, the unaffected or maintained PAM site might execute recurrent editing activity for the same gene target, hence enhancing its on‐target editing efficiency (Alok et al. 2020). Despite the fact that Cas12a has emerged as a suitable candidate for genome editing applications in plant and animal systems, it has certain drawbacks due to the shorter size of its crRNAs (42–44 nt) as compared to Cas9 (140 nt), which can lead to the formation of undesirable secondary structures and a decrease in efficiency (Malzahn et al. 2019). Presently, three Cas12a variants, including AsCas12a, FnCas12a, and LbCas12a, have been demonstrated in a variety of plants, including those with varying efficiencies. Recent optimization of Cas12a orthologs and engineered variants has significantly improved editing efficiency, temperature tolerance, and multiplexing capacity in cereal crops, positioning Cas12a as a complementary system to Cas9 for AT‐rich genomic regions (Zhang, Zhang, et al. 2021; Zhang, Wang, and Li 2021; Zhang, Malzahn, et al. 2021).

### 2.3 Cas13

Cas13 systems enable programmable RNA targeting and have been explored primarily for virus resistance and transcript‐level regulation in plants. While promising for functional studies, Cas13 applications remain largely experimental in cereals and are not yet widely deployed for stable agronomic trait improvement. To date, a wide range of rice genes have been successfully edited using CRISPR‐based technologies for diverse agronomic traits such as yield, stress tolerance, grain quality, plant architecture, and flowering time. A summary of representative studies is provided in Table 3.

**TABLE 3:** CRISPR/Cas based trait improvements in rice.

Target gene(s)	Trait improved	Editing strategy	Key outcome	References
*OsSAP*	Drought tolerance	SDN‐1 knockout	Improved survival and yield stability under drought	Park et al. (2022)
*GW2*	Grain weight & nutrient accumulation	SDN‐1 knockout	Increased grain weight and mineral content	Achary and Reddy (2021)
*OsALS* (base edited)	Herbicide tolerance	SDN‐2 base editing	Precise amino acid substitution conferring resistance	Kuang et al. (2020)
*OsERA1*	Drought tolerance	SDN‐1 knockout	Enhanced ABA sensitivity and water‐use efficiency	Ogata et al. (2020)
*OsSWEET14* (promoter)	Bacterial blight resistance	SDN‐1 promoter editing	Durable resistance without yield penalty	Ponnurangan et al. (2026)
*OsNramp5*	Low cadmium accumulation	SDN‐1 knockout	Reduced Cd in grains with stable yield	Tang et al. (2022)
*OsWaxy* (*GBSS*)	Grain quality (amylose)	SDN‐1 knockout	Improved cooking quality and starch composition	Mahmuda et al. (2021)
Multiplex stress regulators	Stress resilience	Multiplex SDN‐1	Coordinated stress tolerance with minimal trade‐offs	Riaz et al. (2025)

### 2.4 Base Editing

Recent advances in plant base editing have enabled highly efficient and predictable nucleotide substitutions in cereal genomes, allowing precise modulation of enzyme activity and regulatory motifs without inducing double‐strand breaks. Improved cytosine and adenine base editors, including evolved deaminases and optimized Cas variants, have expanded the scope of trait engineering in rice, wheat, and barley. These developments support pathway‐level trait improvement by fine‐tuning enzymatic function, stress signaling, and metabolic fluxes rather than relying on gene knockouts alone (Mishra et al. 2020; Vats et al. 2024).

RNA base editing system consists of REPAIR (RNA editing for programmable A to I (G) replacement) and RESCUE (RNA editing for specific C‐to‐U exchange). RNA base editors (RBE) have recently arisen and been modified to convert A to I in RNA molecules (Abudayyeh et al. 2016; Cox et al. 2017).

Adding the base‐edit repair inhibitor, a glycosylase inhibitor, to the fusion protein and altering the Cas proteins improves editing efficiency or specificity, or both, by incorporating the base‐edit repair inhibitor. Base editing has several advantages over existing CRISPR/Cas technologies and has been successfully applied to plant genomes including those of rice, wheat, maize, potato, watermelon, cotton, tomato, and Arabidopsis (Chen et al. 2017; Hess et al. 2017; Yang et al. 2017; Zong et al. 2018; Tian et al. 2018; Monsur et al. 2020; Shimatani et al. 2017; Li et al. 2018). Base editors can also disrupt genes in plants by introducing premature stop codons or triggering transcript mis‐splicing (Veillet et al. 2019).

Recent advancements have expanded CRISPR‐mediated editing in barley, where several target genes have been manipulated to improve agronomic traits such as nutrient use efficiency, seed dormancy, and vitamin E composition. Representative examples of CRISPR applications in barley are summarized in Table 4, which highlights the target genes, delivery methods, editing efficiencies, and heritability of induced mutations.

**TABLE 4:** List of the genes edited in barley crop with CRISPR technology.

Target gene	Trait improved	Promoter sgRNA/Cas9	No. of gRNA targeted	Selectable marker	Delivery method	Genotype	Explant	Editing frequency (%)	Mutation type	Inheritance	References
*HvMPK6*	Studied the loss of function in gene	2* CaMV35S/ZmUbi1‐int	1	Hygromycine	*Agrobacterium*‐mediated transformation	Golden Promise (GP) and a double haploid line of GP	Immature embryos	Variable	Deletion	—	Křenek et al. (2021)
*HvPDS*	To create mutations in commercial barley verities	U6/ *S*. *pyogenes*	4	Hygromycin resistance cassette PCR based	*Agrobacterium*‐mediated transformation	Golden Promise and four Australian varieties	Anther	53% average	Indel	Heritable mutations in T1 progenies	Han et al. (2021)
*HvARE1*	improvement of nitrogen use efficiency	ZmUbi/CaMV35S	2	Hygromycin resistance cassette	*Agrobacterium*‐mediated transformation	Golden Promise, Legacy, Granger and Stirling	Callus	Variable	Indel	Heritable mutations	Karunarathne et al. (2022)
*HvHPT*, *HvHGGT*	Production of gene knockout mutants to elucidate the genetic control of vitamin E composition in barley	*OsU3/Os UBI10*	4	*hpt*	*Agrobacterium tumefaciens*	Golden Promise	Calli	50%–65%	Indel	Heritable mutations	Zeng et al. (2020)
*Qsd1, Qsd2*	To create mutation in seed dormancy genes	*OsU3/−*	2	PCR based	*Agrobacterium strain* AGL1‐mediated transformation.	Golden Promise	Immature embryos	42.9% and 30%	Indel	Heritable mutations	Hisano et al. (2022)
*HvFT1*	Flowering time regulation and environmental adaptation	U6/maize ubiquitin	2	Hygromycin	*Agrobacterium*‐mediated transformation	Golden Promise	Immature embryos	~40%	Indel	Stable, heritable	Kishchenko et al. (2020)

Being relatively new, base editing tools require additional research to allow the introduction of transversion mutations, while further improving precision, accuracy, and accessibility. With the integration of appropriate Cas variants, CBE and ABE platforms hold great promise for expanding crop improvement strategies in barley and beyond, while minimizing off‐target effects (Mishra et al. 2020).

### 2.5 Prime Editing

**FIGURE 3:**
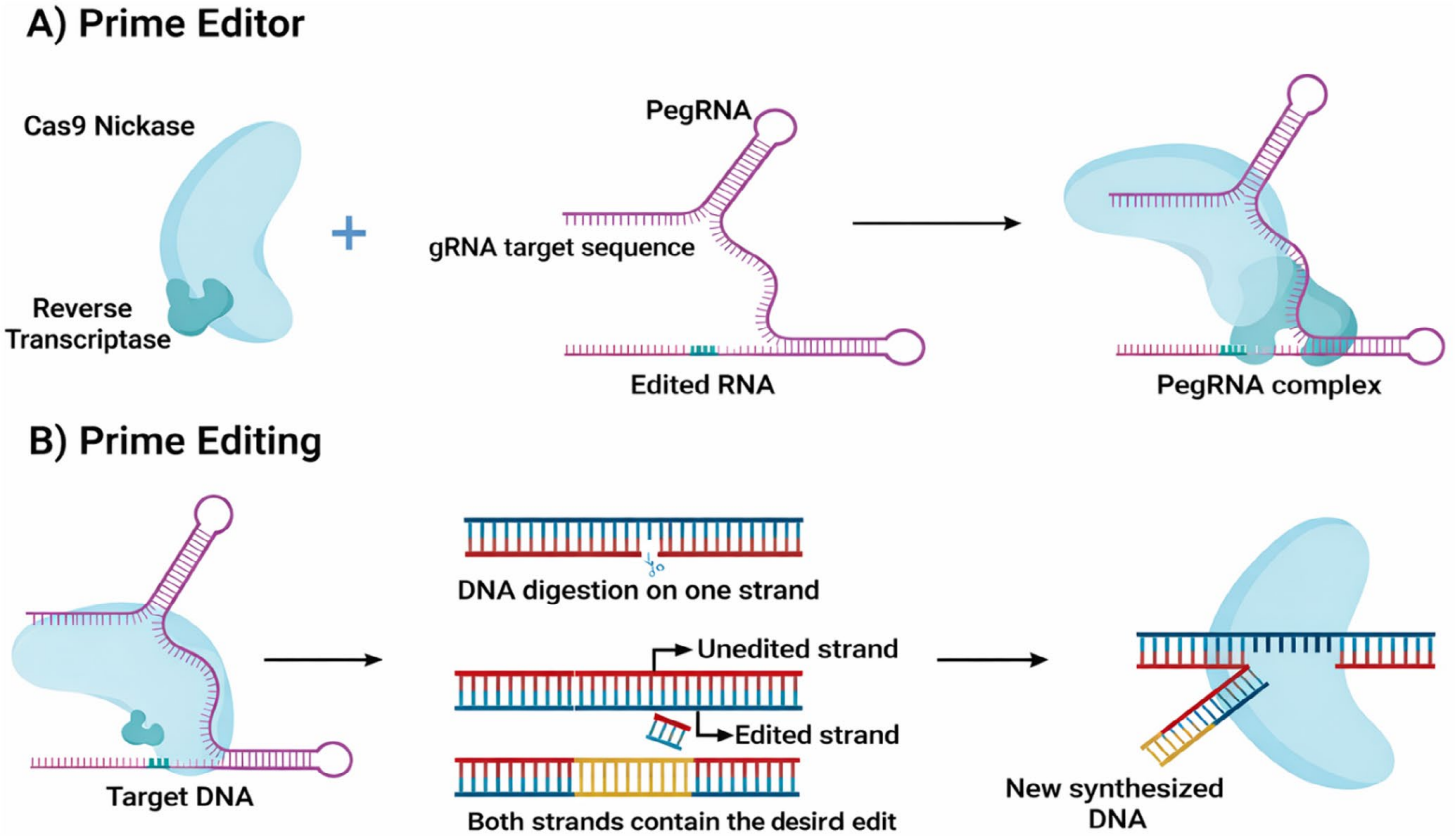
Precise molecular mechanisms orchestrate prime editing, a revolutionary gene‐editing technique (Figure created by the authors using BioRender.com).

Several genome editing approaches remain constrained in their ability to introduce precise nucleotide substitutions or small sequence modifications at predefined genomic loci. Prime editing overcomes these limitations by enabling programmable “search and replace” genome modification without inducing double‐strand breaks or requiring donor DNA templates. The prime editing system comprises three key components: (i) a prime editing guide RNA (pegRNA) that specifies both the target site and the desired edit, (ii) a Cas9 nickase that introduces a single‐strand DNA nick, and (iii) a reverse transcriptase that copies the edited sequence directly into the target locus (Figure 3). Together, these components enable the installation of customizable point mutations and short insertions or deletions with reduced off‐target activity.

Recent advances in plant optimized prime editor architectures including improved pegRNA design, enhanced reverse transcriptase activity, and optimized expression systems have substantially improved editing efficiency and reproducibility in monocot crops. Although prime editing is generally less efficient than base editing for generating transition mutations, it uniquely enables transversion substitutions and complex nucleotide changes that are inaccessible to cytosine and adenine base editors. As a result, prime editing has emerged as a complementary precision tool for cereal genome engineering, particularly for allele replacement and regulatory fine tuning relevant to trait improvement, rather than as a high throughput mutagenesis platform (Vats et al. 2024).

## 3 Delivery Aspect of Genome Editing Components

For the development of novel features in crops without efficient transformation mechanisms, the delivery of genome‐editing components continues to present obstacles. With the developing field of CRISPR and its related technologies, distribution techniques must also evolve. The delivery of genome editing components into plant cells has primarily been restricted to *Agrobacterium* or bombardment. In addition, numerous delivery techniques, including virus‐mediated, RNP‐based, meristem induction, lipofection‐ and PEG‐mediated protoplasts, are routinely employed in plant genome editing applications. However, delivery efficiency remains highly reliant on a number of variables, including explant type, *Agrobacterium* strain, genotype, cassette size, and media variables.

*Agrobacterium*‐mediated transformation, using either a single binary vector or multiple binary vectors introduced into Agrobacterium, has been widely employed to deliver single or multigene expression cassettes into plant genomes. This transformation system is economical, technically straightforward, and broadly accessible across plant biotechnology laboratories. Importantly, *Agrobacterium tumefaciens* mediates the stable integration of transfer DNA (T‐DNA), rather than the entire Ti plasmid, into the plant nuclear genome, enabling the generation of transgenic and genome‐edited plants.

Despite these advantages, *Agrobacterium*‐mediated delivery has several limitations, including constraints on host range and transformation efficiency in certain cereal genotypes, limited capacity for precise control over transgene copy number and insertion sites, and reduced flexibility for delivering preassembled ribonucleoprotein complexes or large DNA constructs. Additionally, simultaneous and independent delivery of multiple editing reagents using separate binary vectors can be technically challenging. To overcome these limitations, alternative direct gene delivery approaches such as particle bombardment (biolistics), protoplast transfection, and emerging nanoparticle‐ and peptide‐assisted delivery systems are increasingly being developed and utilized for plant genome editing applications.

CRISPR/Cas reagents delivered as in vitro transcripts or ribonucleoproteins have enabled DNA‐free genome editing ribonucleoproteins (RNPs). However, this approach can generate several copies of the same gene, resulting in undesired expression changes. The biolistic delivery approach uses DNA‐coated microprojectiles to transfect cells. In contrast, polyethylene glycol (PEG) is used in protoplast transfection to transfer genome editing components into the protoplast following digestion of the cell wall. Using protoplast transfection, ZFNs, TALENs, and CRISPR have been effectively applied for gene knockout investigations in many plant species. The fundamental drawback of this approach is its inability to change all plant species, particularly monocots. The indirect and direct methods of distribution have been widely and effectively utilized in a variety of crops. However, they are limited in terms of crop application, precision, and duration. Therefore, fresh methods must be developed to achieve application on a larger range of crops in the shortest possible time.

Recent technologies, like nanoparticles (NPs) as delivery vehicles, have gained momentum since they may be tailored to the target tissue and organism. Cas9 RNPs, which include negatively charged gRNA molecules, create an instantaneous combination with cationic lipids. Recently, nucleic acids have been utilized as polymeric delivery substrates for Cas9 RNP. However, additional modification of the polymeric coating is required to prevent breakdown by biological pathways and enzymes. Alternatives to encapsulation include protein and nucleic acid modification. Cell‐Penetrating Peptides (CPPs) are short peptide sequences that can easily pass through the cell membrane. They can be coupled with Cas9 protein and gRNA to increase delivery efficiency. However, these peptides do not protect the protein within the cell from protease breakdown and can be combined with other delivery techniques. In addition, nuclear localization sequences (NLSs), which are sequences generated in the cytoplasm for labelling and transporting proteins into the nucleus, are being investigated. They are composed of poly‐arginine/lysine and function as signal molecules coupled to transport proteins in the nucleus. As Cas9 must be delivered into the nucleus, NLSs are effective delivery agents that can be synthesized into proteins or encoded into the Cas9 construct. While the majority of these techniques are still widely used, they have deficiencies that render them inappropriate for effective editing.

Using procedures based on tissue culture, plants must regenerate from altered cells/explants. Consequently, such operations are extremely time‐consuming. Also, transformation techniques are genotype‐dependent, and viable protocols for resistant crop species have not been created. Consequently, a new methodology has been developed that eliminates the requirement for standard tissue culture procedures and in planta methods such as floral dipping (Ji et al. 2020). Using genome‐editing machinery, gene‐edited somatic cells are reprogrammed into meristematic cells via expressions of developmental regulator (DR) genes, such as *WUSCHEL2 (Wus2)* and *BABY BOOM*. In Arabidopsis, the ectopic expression of DRs such as Wus2, SHOOT MERISTEMLESS (STM), and MONOPTEROS (MP) stimulates the formation of meristem‐like structures. In addition, the co‐expression of Wus/STM and CRISPR/Cas9 cassette in *Nicotiana benthamiana* was used to target the phytoene desaturase (*PDS*) gene. Later, the meristems develop into shoots. (Maher et al. 2020).

The mutation frequency of these *PDS* mutant plants was comparable to that of *Agrobacterium*‐transformed plants, according to a comparison study. For the induction of the root system, the shoot‐induced genome‐edited plants were transplanted straight into the soil or a rooting medium, where they eventually developed into full plants. In a separate investigation, CRISPR/Cas9 expressing soil‐grown plants were injected with Agrobacterium cultures containing the appropriate DR and sgRNA at the areas where meristems had been excised. The in‐planta transformation procedure successfully produced abundant shoot development. Co‐expression of DRs and the CRISPR/Cas9 system resulted in the modification of developmental phases of altered somatic cells, stimulation of meristem development, and their growth in fertile plants. This technique obviated the necessity for tissue culture processes in order to get genome‐edited plants. Exclusion of tissue culture‐based genome editing decreases expense, labour, and increases productivity. De novo meristem induction‐mediated genome editing is an emerging field of study. Moreover, aberrant growth has been seen as a result of the constitutive expression of DRs. This can be circumvented through the inducible expression of DRs. While de novo shoot meristem induction has been utilized in grape, potato, and tomato crops, its applicability in plants with heritable mutations has not yet been investigated. This technique's applicability must also be extended to main food crops (Chennakesavulu et al. 2021; Ji et al. 2020).

Expansion of in planta transformation in genome editing techniques such as base editing would also aid in crop breeding's realization of its full potential (Ji et al. 2020). As a tissue‐culture‐free technique, the infection of meristematic tissues with a virus expressing SpCas9 protein has also been accomplished. Tobacco rattle virus (TRV) was employed for this purpose; the gRNA was fused with Arabidopsis *Flowering Locus T (FT)* mRNA in tobacco, and the resultant edited plant exhibited mutations in 65%–100% of its offspring. Further, TRV was not detected in the offspring, so protecting them from any external effects of the virus. This in planta gene‐editing approach induced minor alterations in a gene with great success, indicating its immense potential. As TRV was solely optimized for tobacco, it demonstrated significant deficiencies, such as the determination of species‐specific efficient viral vectors. Additionally, the mobility of gRNA fused with Arabidopsis FT should be tested in different plants, as the editing efficiency is dependent on translocation ability.

### 3.1 Improving Transformation Efficiency Across Species and Genotype

Although genome editing has emerged as a transformative approach for improving essential food crops, substantial practical barriers continue to limit its widespread application. Unlike animal genome editing, plant genome editing requires efficient delivery of editing reagents into plant cells, reliable selection of edited events, and regeneration of complete, fertile plants carrying heritable modifications. Despite major advances in CRISPR/Cas technologies, transformation and regeneration efficiencies remain highly genotype and species dependent for many crops, particularly cereals, and thus represent a critical bottleneck for translating genome editing into breeding pipelines (Maher et al. 2020; Gao and Zhao 2021).

Recent studies have demonstrated that transient expression of developmental regulator (DR) genes, such as *BABY BOOM* (*BBM*) and *WUSCHEL2* (*WUS2*), in combination with optimized phytohormone regimes, can significantly enhance plant transformation efficiency. Expression of these regulators reprograms somatic cells toward meristematic competence, enabling de novo shoot formation and regeneration of edited plants without prolonged tissue culture steps (Maher et al. 2020; Lowe et al. 2016). While this strategy has shown promise in selected crop species, its broader applicability across diverse cereal genotypes remains limited, and further refinement is required to ensure developmental stability, heritable transmission of edits, and compatibility with advanced genome editing modalities such as base and prime editing.

Overall, transformation efficiency remains the principal constraint in applying genome editing for crop improvement. Although transient transformation systems are sufficient for functional genomics studies, stable transformation enabling precise, single copy integration and consistent regeneration is essential for breeding‐oriented applications. Continued innovation in genotype‐independent transformation and regeneration technologies will therefore be crucial for accelerating the adoption of CRISPR‐based precision breeding in agriculture (Zaidi et al. 2016; Cardi et al. 2023).

## 4 Precise Genome Editing in Cereals Using CRISPR/Cas9 Approach

Precise genome editing in cereals using the CRISPR/Cas9 system has rapidly advanced, providing opportunities for targeted trait improvement beyond the limits of conventional breeding. In rice, wheat, barley, and maize, CRISPR/Cas9 has been employed to enhance yield‐related traits, improve grain quality, and confer tolerance to both biotic and abiotic stresses. Several studies have also demonstrated the generation of transgene‐free edited lines, which enhance prospects for biosafety and commercialization.

Recent advances in CRISPR‐based genome editing have shifted cereal improvement from single‐gene mutagenesis toward pathway‐informed precision breeding, integrating multiplex editing, regulatory region engineering, and high‐resolution base and prime editing approaches to modulate complex agronomic traits (Zaidi et al. 2016; Cardi et al. 2023; Vats et al. 2024). The following table (2–4) summarizes the major achievements of genome editing in key cereal crops, highlighting the specific genes edited, targeted traits, and outcomes reported.

### 4.1 Grain Yield Architecture and Source Sink Regulation

Genes targeted for yield improvement through CRISPR/Cas9 generally encode enzymes or regulators that control key metabolic nodes rather than yield itself. Yield gains occur only when such edits shift carbon flux, hormone turnover, or assimilate partitioning in favor of reproductive growth. Therefore, the following examples are discussed in terms of their metabolic consequences rather than as single‐gene yield determinants.

At the metabolic level, CRISPR‐mediated yield improvement is primarily achieved through reprogramming hormone‐regulated enzymatic processes that govern meristem activity and assimilate allocation. For instance, editing of cytokinin oxidase/dehydrogenase (CKX) genes reduces cytokinin degradation, increasing active cytokinin pools in inflorescence meristems. This enzymatic shift enhances cell division rates and prolongs meristem activity, resulting in increased grain number. Similarly, targeted modification of gibberellin biosynthesis genes such as GA20‐oxidases alters the flux through the gibberellin pathway, reducing excessive stem elongation and redirecting carbon resources toward reproductive tissues. These hormone‐mediated metabolic adjustments collectively improve source‐sink balance and grain yield potential.

Grain yield in cereals is a complex quantitative trait influenced by multiple genetic components, regulatory networks, and environmental interactions, making it an ideal target for multiplex and regulatory‐region genome editing rather than single‐gene modification. Also, these techniques greatly assisted breeders in detecting allelic variations for crop improvement. However, with the complexity of the trait governed by multiple genes and regulatory networks, there is a demanding rapid way to elucidate gene functions and trait improvement. An important agronomic trait is known to be firmly controlled by negative regulators. Negative regulators encoding gene(s) can be knocked out or expression can be down regulated by modifying the upstream promoter region (Zhang, Zhang, et al. 2021). Using CRISPR/Cas9, simultaneous knockout of multiple negative regulators such as grain width 2 (*GW2*), grain width 5 (*GW5*), and thousand‐grain weight 6 (*TGW6*) significantly increased grain yield in rice (typically achieved through SDN‐1 editing) (Zhang, Massel, et al. 2018). Recent multiplex CRISPR/Cas9 studies demonstrate that coordinated editing of negative regulators controlling grain size, meristem activity, and hormone turnover can enhance yield by reprogramming source‐sink relationships rather than acting through single gene effects. Editing cytokinin degradation pathways, grain‐size regulators, and regulatory regions controlling meristem determinacy has resulted in measurable increases in grain number and yield across cereal species (Liu et al. 2021; Sourdille et al. 2025).

Gene controlling panicle length (*OsPIN5b*) was targeted in rice using the CRISPR. The CRISPR mutant rice lines exhibited better yield. Two other stress‐related genes were targeted along with *OsPIN5b* simultaneously. This study proved the application of CRISPR/Cas9 in editing multiple genes controlling different traits in plants. Knocking out undesirable regulatory genes and factors can improve the overall plant architecture which can increase the cereal yield significantly (Ahmad et al. 2021).

The CRISPR/Cas9 modification of *OsLOGL5* increases root growth, a number of tillers, and yield of rice. Cytokinin hormonal regulator protein *OsLOGL5* is important for yield increase in rice. CRISPR/Cas9 editing of *OsLOGL5* increased root growth, tiller number, and grain yield in rice by enhancing cytokinin‐mediated meristem activity (Wang et al. 2016). In addition to grain quality, size, and plant architecture, improvement of photosynthesis based on CRISPR/Cas9 editing holds promises in yield enhancement in cereal crops. CRISPR/Cas9 can be useful in manipulating the expression of the gene encoding negative regulators of photosynthesis. It was reported that knocking out NRP1, a negative regulator and used to knock out transcription factor (TF), NEGATIVE REGULATOR OF PHOTOSYNTHSIS 1 (NRP1), increases photosynthesis and biomass production in edited rice plants (Chen et al. 2021). As photosynthesis determines crop yield and growth, manipulating related genes holds promises of desired changes. Ribilose‐1,5‐biphospate carboxylase/oxygenase (Rubisco) is considered the preferred and a prime target for modulating photosynthetic efficiency in plants. CRISPR‐Cas9 mediated knock‐out of RbcS (Rubisco small subunit) was performed in C3 rice (Matsumura et al. 2020). Subsequently, the replacement of rice RbcS with sorghum (C4) RbcS increased the photosynthetic efficiency. CRISPR/Cas9 edited rice plants also showed increased crop productivity under high CO_2_ conditions when compared to wild type rice plants (Matsumura et al. 2020).

On the other hand, in cis‐regulatory regions, promoter editing is proved to be a promising way to modify yield‐related traits in cereal crops. CRISPR is a perfect tool for altering promoter regions, to fine‐tune gene expression, so as to understanding the factors such as seed dormancy, grain number and seed dispersal, which are responsible of for the developmental shifts in major cereal crops. Genes encoding WD40, KRN2, and *OsKRN2* were edited in maize and rice, resulting in increased grain number and kernel row number, with reported yield gains of up to 10% in maize and 8% in rice (Wenkang et al. 2022). This study adopted the CRISPR knock‐out technique to create variations within the non‐coding upstream region of KRN2 (kernel row number2) genes. Negative regulators like KRN2 which are known to control grain number and panicle branching are a potential target for crop improvement (Wenkang et al. 2022). In Maize, promoter regions of *CLR* genes are associated with *CLAVATA‐WUSCHEL* pathway. Weak alleles were created by using CRISPR/Cas9 in maize to increase the yield‐related traits (Liu et al. 2021).

### 4.2 Drought Tolerance as a Yield‐Stabilizing Trait

Drought tolerance is not a yield‐enhancing trait per se but a critical yield‐stabilizing attribute that preserves productivity under water‐limited conditions. In cereal crops, drought primarily reduces yield by disrupting photosynthesis, impairing carbon assimilation, accelerating senescence, and limiting assimilate transport to developing grains. CRISPR/Cas‐mediated genome editing has enabled precise modification of key regulatory nodes within drought‐responsive metabolic and signaling pathways, thereby mitigating these negative effects and stabilizing yield across environments.

At the enzymatic and metabolic levels, many CRISPR‐based drought tolerance strategies target the abscisic acid (ABA) signaling pathway, which integrates water stress perception with downstream physiological responses. Editing of ABA receptor genes belonging to the *PYR/PYL* family alters the interaction between ABA, protein phosphatase 2C (PP2C), and SNF1‐related protein kinases (*SnRK2s*). These modifications fine‐tune stomatal aperture regulation, reducing transpirational water loss while maintaining sufficient CO_2_ influx for photosynthesis. Concurrently, downstream activation of antioxidant enzymes and osmoprotectant biosynthesis pathways reduces oxidative damage under drought stress, preserving cellular metabolic integrity.

CRISPR‐mediated modification of drought‐responsive transcription factors and negative regulators further contributes to yield stability by maintaining carbon fixation and assimilate flow during stress episodes. For example, editing genes that repress stress‐responsive metabolic pathways enhances the expression of enzymes involved in osmotic adjustment, reactive oxygen species scavenging, and cellular energy balance. These changes sustain photosynthetic efficiency and delay stress‐induced senescence, ensuring continued carbohydrate availability for grain filling.

Importantly, drought tolerance edits indirectly support yield by stabilizing source‐sink relationships rather than increasing yield potential under optimal conditions. By preserving photosynthetic activity, maintaining leaf function, and sustaining assimilate transport to reproductive tissues, CRISPR‐engineered drought‐tolerant lines exhibit reduced yield penalties under water scarcity. These outcomes highlight that effective drought tolerance arises from coordinated reprogramming of signaling networks and metabolic pathways rather than from single‐gene effects.

Overall, CRISPR/Cas‐based genome editing offers a powerful approach to enhance drought resilience in cereals by targeting regulatory and enzymatic components of stress perception and metabolic adaptation. Such pathway‐level interventions are essential for maintaining yield stability in the face of increasing climatic variability and are most effective when integrated with complementary edits in yield architecture and carbon metabolism pathways. Recent pathway‐level genome editing studies further confirm that drought tolerance functions primarily as a yield‐stabilizing trait rather than increasing yield potential under optimal conditions (Li, Lin, and Zhang 2022; Lola et al. 2023).

### 4.3 Nutrition Fortification

Grain quality traits modified through CRISPR/Cas9 are directly linked to enzymatic steps in starch biosynthesis and carbon partitioning during grain filling. Targeted editing of starch branching enzymes (SBEI and SBEIIb) alters the degree of amylopectin branching, shifting carbon flux toward increased amylose and resistant starch formation (representing SDN‐2‐type modifications). Similarly, modification of granule‐bound starch synthase (*GBSS/Waxy*) genes affects glucan elongation dynamics, influencing starch structure, grain density, and end‐use quality. These enzymatic modifications not only enhance nutritional value but also impact grain weight and filling efficiency, demonstrating the interconnected nature of quality and yield traits.

Improving food quality traits such as nutritive value and storage quality can be effectively targeted using CRISPR/Cas9 mediated genome editing. Targeted modification of starch biosynthetic genes, including *Waxy* and starch branching enzymes, has enabled the development of cereal lines with altered amylose composition and enhanced resistant starch content (Sun et al. 2017). Recent multiplex CRISPR/Cas9 editing of starch branching enzyme genes in rice significantly increased resistant starch levels, demonstrating precise metabolic reprogramming of endosperm starch biosynthesis with nutritional benefits (Biswas et al. 2023; Qi et al. 2020). Similarly, CRISPR/Cas9 mediated editing of starch branching enzymes in maize generated high‐amylose and resistant‐starch phenotypes without compromising agronomic performance, highlighting the translational potential of genome editing for cereal nutritional improvement (Ma et al. 2024). These studies exemplify how CRISPR‐based modulation of starch biosynthesis pathways can generate cereals with improved dietary value. In addition to starch quality, lignocellulosic composition has also been targeted for value enhancement. Targeted CRISPR/Cas9‐induced mutation of the cinnamyl alcohol dehydrogenase gene (*OsCAD2*) in rice reduced lignocellulose recalcitrance, thereby improving biomass saccharification efficiency and expanding downstream utilization potential (Zhang, Wang, and Li 2021).

These findings further confirm the utility of CRISPR/Cas9 for precise manipulation of starch biosynthetic enzymes to enhance nutritional quality in cereal crops. These nutritional improvements are underpinned by targeted modification of key biosynthetic enzymes, demonstrating how CRISPR enables precise metabolic engineering of grain composition without compromising yield.

### 4.4 Biotic Stress Resistance

Biotic stress resistance is one of the most successful applications of CRISPR/Cas9 in cereal crops, particularly through editing of susceptibility (S) genes and pathogen effector‐binding sites. Unlike traditional resistance breeding, genome editing allows precise disruption of host susceptibility pathways while minimizing yield penalties. Recent studies increasingly combine disease resistance edits with yield and architecture‐related traits, underscoring the importance of integrated trait improvement rather than isolated resistance engineering. Advanced CRISPR‐based strategies, including multiplex and precision genome editing, enable targeted modification of host susceptibility and resistance pathways, facilitating durable disease resistance while minimizing associated yield penalties.

Editing of disease susceptibility (S) genes has emerged as one of the most effective genome editing strategies to confer durable resistance against bacterial and fungal pathogens in cereal crops (Li, Jiao, et al. 2022). Recent CRISPR/Cas‐based studies demonstrate that targeted modification of host susceptibility pathways can block pathogen exploitation of host metabolism while minimizing trade‐offs associated with constitutive defense activation (Tyagi et al. 2021; Yin and Tsai 2025). In rice, precise editing of SWEET sucrose transporter genes and their promoter regions has been shown to disrupt pathogen‐induced sugar efflux, thereby limiting carbon availability to *Xanthomonas oryzae* and enhancing resistance to bacterial blight without major yield penalties (Varshney et al. 2019; Manzoor et al. 2024). These studies confirm that metabolic deprivation of pathogens through host gene editing represents a robust resistance mechanism.

Similarly, CRISPR‐mediated editing of *MLO* susceptibility genes remains a cornerstone strategy for powdery mildew resistance in wheat and other cereals. Recent work has refined this approach by combining *TaMLO* knockouts with compensatory metabolic or transporter gene modulation to mitigate pleiotropic effects traditionally associated with *mlo*‐based resistance (Abdelrahman et al. 2021; Sourdille et al. 2025). In particular, multiplex CRISPR strategies that ectopically regulate tonoplast sugar transporters or fine‐tune defense‐related carbohydrate partitioning have successfully uncoupled disease resistance from growth penalties, resulting in mildew‐resistant wheat lines with stable yield performance (Abdelrahman et al. 2021; Yin and Tsai 2025). Beyond susceptibility transporters, CRISPR/Cas9 based disruption of negative regulators of immunity, including ethylene‐responsive transcription factors and other signaling repressors, has further expanded the toolbox for engineering disease resistance in cereals. Recent rice studies demonstrate that editing transcriptional repressors within defense signaling networks enhances blast resistance by strengthening basal immunity while preserving photosynthetic and metabolic efficiency (Manzoor et al. 2024; Tyagi et al. 2021). Collectively, these findings underscore that modern CRISPR‐enabled disease resistance in cereals is increasingly pathway‐aware, integrating susceptibility gene editing with metabolic compensation to achieve durable resistance without yield compromise.

From a metabolic perspective, biotic stress resistance achieved through CRISPR editing protects yield by preventing pathogen‐induced diversion of host carbon resources. Editing susceptibility genes such as SWEET sugar transporters disrupts pathogen‐triggered sucrose efflux from host cells, thereby retaining carbohydrates for host metabolism and grain development. Similarly, knockout of MLO genes reduces pathogen compatibility without extensive activation of defense‐associated energy sinks. These modifications illustrate how disease resistance traits preserve metabolic efficiency and carbon availability, indirectly sustaining yield under pathogen pressure.

Targeted editing of host susceptibility factors has also been successfully applied to confer resistance against viral pathogens in cereal crops. This method has been used in dicot plant systems previously. In rice, the susceptible (*S*)‐type allele of the initiation factor 4 gamma (*eIF4G*) gene was targeted to edit using the CRISPR/Cas9. Novel *eIF4G* alleles generated in rice cultivars using CRISPR/Cas9 decreased the susceptibility to Rice tungro disease (RTD) caused by Rice tungro spherical virus. This study is the right example to target *S* gene editing to improve virus resistance in cereal crops. Targeted editing of *SbCCD7, SbCCD8, SbMAX1*, and a *SbDUF*‐domain gene within the LGS1 locus has led to the development of sorghum lines resistant to the parasitic weed *Striga hermonthica* (Kaniganti et al. 2025) (consisting of SDN‐3‐type interventions). The following section integrates the trait‐specific examples discussed above to illustrate how CRISPR‐mediated gene edits converge on common enzymatic and metabolic pathways governing cereal yield and resilience.

### 4.5 Enzyme and Pathway‐Level Basis of CRISPR‐Mediated Trait Improvement in Cereals

Improvement of complex agronomic traits such as yield, stress tolerance, and grain quality ultimately depends on modifications of enzymatic activities and metabolic pathways (Yin and Tsai 2025). CRISPR/Cas‐mediated genome editing enables precise manipulation of genes encoding enzymes, regulatory proteins, and transcriptional controllers, thereby reshaping metabolic fluxes and physiological processes in cereals.

#### 4.5.1 Starch Biosynthesis and Carbon Partitioning

Grain yield and quality in cereals are strongly influenced by starch biosynthesis enzymes, including ADP‐glucose pyrophosphorylase (AGPase), starch branching enzymes (SBEI and SBEII), granule‐bound starch synthase (GBSS/Waxy), and soluble starch synthases. CRISPR‐mediated editing of Waxy (GBSS) reduces amylose synthesis by limiting glucan chain elongation, altering starch structure and improving cooking quality without yield penalties. Similarly, knockout of SBEIIb increases resistant starch by modifying branch point frequency, redirecting carbon flux toward amylose accumulation. These enzymatic alterations directly influence endosperm metabolism, grain filling rate, and final grain weight.

#### 4.5.2 Hormone Biosynthesis and Signal Transduction

Plant hormones regulate yield‐related traits through enzymatic control of biosynthesis and degradation pathways. Editing of cytokinin oxidase/dehydrogenase (CKX) genes increases cytokinin availability by reducing enzymatic degradation, enhancing meristem activity, panicle branching, and grain number. Mutations in gibberellin biosynthesis enzymes (e.g., GA20ox) reduce excessive stem elongation by limiting GA synthesis, improving lodging resistance while reallocating assimilates to reproductive tissues. CRISPR‐based modulation of ABA receptors (PYL proteins) alters downstream phosphatase and kinase activities, improving drought resilience and stabilizing yield under water‐limited conditions.

#### 4.5.3 Photosynthesis and Primary Metabolism

Yield improvement is closely linked to photosynthetic efficiency and carbon fixation capacity. CRISPR‐mediated editing of negative regulators of photosynthesis, such as NRP1, enhances Rubisco activation state and electron transport efficiency. Replacement or modification of Rubisco small subunits (RbcS) alters enzyme kinetics, improving CO_2_ fixation efficiency and biomass accumulation. These enzymatic enhancements increase source strength, supporting greater assimilate availability for grain filling.

#### 4.5.4 Nitrogen Assimilation and Protein Metabolism

Nitrogen use efficiency is governed by enzymes involved in nitrate uptake, assimilation, and amino acid biosynthesis. Editing of genes regulating asparagine synthetase (ASN) reduces free asparagine accumulation in grains by altering nitrogen storage metabolism, improving grain quality and reducing acrylamide formation without compromising yield. CRISPR‐mediated modification of nitrogen‐responsive regulators also optimizes enzymatic coordination between carbon and nitrogen metabolism, contributing to stable productivity.

#### 4.5.5 Defense‐Related Enzymes and Metabolic Trade‐Offs

Biotic stress resistance often involves activation of defense‐related enzymes, including cell wall‐modifying enzymes, sugar transporters, and secondary metabolite biosynthetic enzymes. Editing of SWEET sugar transporter promoters restricts pathogen‐induced sucrose efflux, depriving pathogens of carbon resources without disrupting host metabolism. Knockout of MLO genes alters cell wall‐associated enzymatic pathways, strengthening basal defense while minimizing metabolic costs when combined with compensatory edits. These targeted interventions mitigate the yield penalties traditionally associated with defense activation.

Collectively, these examples illustrate that CRISPR/Cas‐mediated trait improvement in cereals operates by fine‐tuning enzyme activities and metabolic pathways rather than introducing entirely new functions. Such pathway‐aware editing enables rational trait stacking, allowing yield enhancement, stress tolerance, and grain quality to be improved simultaneously.

## 5 Challenges in Genome Editing of Cereals

Due to its versatility and use in numerous genome editing applications, CRISPR has emerged as one of the most versatile genetic engineering techniques in recent years. Genome editing techniques are more precise, quicker, and less expensive than conventional methods and transgenic technologies in achieving targeted crop improvement. Still, genome editing confronts numerous obstacles in its application, and in order to improve its applicability in cereals and other crops, these obstacles must be overcome in order to support the effective implementation of these genome editing techniques for crop development with long‐term prospects. There have been a number of key difficulties to overcome, including regulatory concerns and social acceptance of genome‐edited crops, both of which are important factors in the commercialization of genome edited crops.

The first and most significant problem with this approach is developing an effective delivery mechanism for getting the genome editing reagent into plant cells while avoiding species and genotype‐dependent transformation. The transformation efficiency, which must be tuned for different cultivars, is one of the many obstacles that the CRISPR‐based genome editing approach faces. Although transformation techniques have been devised for most cereal crops, they are usually genotype specific. Various techniques, including protoplast transfection, *Agrobacterium*‐mediated transformation, and particle bombardment, have been employed, each with inherent limitations related to efficiency, genotype dependence, and regeneration capacity (Su et al. 2023). However, many species or variations, primarily wild relatives of major crops, orphan crops, and non‐crop species with great nutritional potential, still lack reliable transformation procedures. Genome editing cannot be explored effectively in the genetic background of high yielding commercial cultivars in such a setting. Other minor difficulties include the requirement of PAM (for many Cas variations), which may make genome editing for a gene lacking the specific PAM sequence difficult. Furthermore, many elite cereal types have low regeneration capabilities and hence resist transformation.

Regulatory frameworks and public acceptance for genome edited crops vary worldwide, with some countries adopting product‐based approaches that exempt certain genome edited plants from stringent GMO rules while others maintain GMO equivalent oversight, profoundly influencing commercialization, trade, and societal acceptance (ISAAA 2019; Buchholzer et al. 2023; Fernández Ríos 2025). Recently, in India a separate regulatory process has been introduced for such technologies that takes them out of the purview of the Genetic Engineering Appraisal Committee or GEAC.

The next difficulty is to have a better understanding of the complicated signaling networks that control the primary attribute. This will demonstrate how genes and their interactions with the environment have an impact. Many crops' critical genes have remained unknown, despite the fact that molecular approaches to genetic enhancement might be extremely efficient. Precision editing with multiplexing is the next issue, despite ongoing advancements in genome editing technologies to achieve efficient base editing. However, in practice, only a small number of sgRNAs (less than 10) have been expressed in plants.

Genome editing has evolved into the most important biotechnological tool, contributing significantly to the advancement of biological knowledge and the field of biotechnology as a whole, allowing for rapid advances in industry, health, and agriculture. There has been rapid progress in the development of CRISPR‐based genome editing technologies over the past 10 years; these have been used in a variety of domains, such as crop improvement and plant functional genomics. Examples of genome editing technologies in plants include the engineering of sequence‐specific nucleases, delivery of editing reagents, generation and selection of editing events, as well as characterization and use of intact plants. These technologies have an impact on the public's acceptance and regulatory approval of genome‐edited plants. However, there is still a need for more research into the molecular and genetic mechanisms of genome editing, as well as ongoing advances and creative applications of genome editing technologies in plants. Plant genome editing presents significant obstacles, as detailed below.

Polyploidy is defined as the acquisition of one or more full chromosomal sets by an organism. Polyploidy can be divided into three categories: autopolyploids, allopolyploids, and segmental allopolyploids. In many ways, polyploidy genomes are complicated. They are challenging to sequence because of repetitive sequences and a larger genome size. The increased number of gene copies and the different functionalities of duplicated genes that have developed as a result of neofunctionalization make functional annotations extremely challenging. The complexity makes obtaining the required mutations challenging. Some mutations, most notably knockdown or knockout mutations of genes, may not modify the phenotype due to the dosage effect of extra paralogous copies of the genes. Such challenges arise when trying to modify the DNA of a polyploid crop like wheat.

In polyploid cereals such as wheat, the presence of multiple homoeologous gene copies poses a significant challenge for precise genome editing, as functional redundancy often masks phenotypic effects unless all homoeologs are simultaneously modified (Rey et al. 2017). Recent studies demonstrate that CRISPR/Cas9 can be used to edit all homoeologs of a target gene in polyploid wheat, but achieving complete knockout across A, B, and D genomes remains technically demanding and often requires optimized multiplexing strategies to increase editing efficiency (Errum et al. 2023; Komura et al. 2025).

## 6 Addressing Technical, Regulatory, and Societal Barriers in Plant Genome Editing

Only by fine‐tuning the gene or genetic element of interest in agricultural plants can genome editing's immense potential in agriculture be realized (Kwon et al. 2019; Oliva et al. 2019). However, due to a lack of understanding of biological processes including genes, pathways, networks, and their interactions with environmental factors, the number of candidates for crop enhancement by genome editing is currently restricted. CRISPR‐based genome editing technologies, on the other hand, are beginning to demonstrate the feasibility of undertaking genome‐wide and high‐throughput functional genomics, speeding up gene/trait discovery in model and non‐model crops (Lu et al. 2017; Meng et al. 2017). The task is to combine multidisciplinary approaches to find targets that have the potential to produce favorable agronomic features while also being amenable for genome editing (Araus et al. 2018).

From a regulatory perspective, genome edits classified as SDN‐1 and SDN‐2 are often treated differently from SDN‐3 modifications in several jurisdictions, which has important implications for the approval and commercialization of genome‐edited cereal crops.

### 6.1 Base Replacement With High Efficiency

Most plant genome editing focuses on targeted mutagenesis, while base replacement remains limited due to low efficiency in many crops. Since key agronomic traits often arise from SNPs, precise nucleotide substitution via HDR or prime editing holds great potential. Prime editing offers advantages over conventional base editing by enabling targeted replacement of small genomic regions, but further optimization is needed for broad application in diverse plant species (Anzalone et al. 2019; Rees and Liu 2018).

### 6.2 Advancing Public Acceptance and Regulatory Governance of Agricultural Genome Editing

Due to the ability of genome editing technologies to produce genotypic and phenotypic variations in plants that are indistinguishable from those obtained naturally or through conventional mutagenesis methods, most regulatory regimes do not readily classify genome edited products as genetically modified organisms (GMOs) (Wolt et al. 2016). Genome editing can speed up and improve crop breeding, increase genetic production potential, create germplasm that is more resistant to pests, diseases, and other abiotic stresses, and lengthen the shelf life of food products to cut down on food waste. Finding genome editing to be more socially and legally acceptable than transgenic approaches would therefore be a challenge. It's critical to build public trust and influence regulatory frameworks, which will have an impact on the utility of genome editing in agriculture, and this requires increasing transparency, information sharing, and openness about genome editing techniques and uses.

### 6.3 Cas9 Off‐Target Effect and Improved Cas9 Variant

The main concern with CRISPR/Cas9‐mediated genome editing is Cas9's off‐target effect (Saini, Devrani, et al. 2025). It has the potential to produce unintended genomic cleavage. Toxicity at the cellular level might result from a higher number of off‐target events, which is not good for plants. In addition, the chromosomal rearrangements induced by the repair of these off‐target DSBs, including deletions, inversions, and translocations, might be damaging to plants (Brunet and Jasin 2018; Lee et al. 2012; Kweon et al. 2013). Plants, on the other hand, have been observed to have minimal off‐target activity when compared to animals. Cas9's specificity is defined by the 20‐nucleotide sgRNA guide sequence and the PAM sequence.

Many studies have found off‐target DNA cleavage in sgRNA sequences with 1–5 bp mismatches. PAM sequence is said to have a role in Cas9 binding, and while the 3′ end is important for target identification, R‐loop creation, and nuclease activity activation in Cas9, minor mismatches at the 5′ end are tolerated (Sander and Joung 2014). The composition and structure of guide RNA are the two most important parameters determining on‐target and off‐target cleavages. Off‐target events can be reduced by changing the content and structure of sgRNA. This can be accomplished in a variety of ways, including truncation of sgRNA at the 5′ end or 3′ end using sgRNA nickase (Tsai et al. 2015), very low or high GC content makes sgRNA less eventful, and truncation of sgRNA at the 5′ end or 3′ end using sgRNA nickase. The efficient sgRNA can be selected based on the GC content of the seed sequence to carry out CRISPR/Cas9 mediated genome editing with minimum off‐target events (Ren et al. 2014). In silico prediction, T7E1 test, HTGTS, ChIP‐seq, IDLV, fluorescence in situ hybridization, deep sequencing, and other approaches have been published to investigate off‐target events. Digenome‐seq and GUIDE‐seq developed as precise methods for off‐target identification with 0.1% sensitivity among these techniques.

According to the research, rice has a greater rate of persistent mutation transmission in multiple generations utilizing CRISPR/Cas9 than *Arabidopsis* (Vats et al. 2019). When *Arabidopsis* plants were altered with CRISPR/Cas9, it was discovered that somatic mutations in the T1 generation did not carry through to the T2 generation, but germline mutations in the T1 generation were passed on to the T2 and T3 generations as Mendelian inheritance (Feng et al. 2014). These findings show that germline mutations are stably absorbed into subsequent generations. A CRISPR/Cas9 construct has been developed using a pollen‐specific promoter, which resulted in the generation of heritable biallelic T1 mutants (Mao et al. 2016). As a result, utilizing germline specific promoters, it is possible to achieve desired Cas9 expression with increased germline mutation, resulting in sustained transmission in future generations. Cas9 variations have been created to decrease the off‐target effect. The fCas9 by combining dCas9 and FokI nuclease has been developed, and the fCas9‐derived modified cells demonstrated 140‐fold enhanced specificity (Guilinger et al. 2014). When 3–4 amino acids were modified in Cas9, no off‐target effects were observed. Hence, off‐target events can be avoided by replacing these Cas9 versions with Cas9 (Kleinstiver et al. 2016).

### 6.4 CRISPR/Cas Genome Editing Versus Genetic Transformation and Regulatory Provisions

CRISPR/Cas‐based genome editing offers several advantages over conventional genetic transformation approaches used in crop improvement. Traditional transgenic methods rely on random insertion of foreign DNA, often resulting in unpredictable gene expression, insertional mutagenesis, and regulatory complexity. In contrast, CRISPR/Cas enables precise, site‐specific modifications of endogenous genes or regulatory regions, frequently without introducing foreign DNA.

Importantly, CRISPR/Cas can generate transgene‐free plants through transient expression or ribonucleoprotein (RNP) delivery, producing genetic outcomes indistinguishable from natural mutations or conventional mutagenesis. As a result, many regulatory agencies classify certain genome‐edited crops (e.g., SDN1 and SDN2 edits) differently from GMOs. The United States, Japan, Argentina, Brazil, and India have adopted science‐based regulatory frameworks that exempt transgene‐free genome‐edited crops from stringent GMO regulations, significantly accelerating product development and commercialization.

From a breeding perspective, CRISPR/Cas shortens breeding cycles, enables multiplex editing of polygenic traits, and allows direct improvement of elite cultivars without linkage drag. These features collectively make genome editing a superior alternative to traditional genetic transformation for precise, rapid, and socially acceptable cereal crop improvement.

## 7 From Lab to Market: CRISPR‐Edited Crops, GMOs, and the Ethical Barriers Ahead

Targeted genome editing has emerged as an efficient alternative to conventional breeding and transgenic approaches for crop improvement (Belhaj et al. 2013). Among genome editing tools, CRISPR has surpassed first‐generation nucleases such as meganucleases, TALENs, and ZFNs due to higher precision and reduced off‐target effects (Gohil et al. 2021; Saini et al. 2023; Saini, Semwal, et al. 2025). Originating from the prokaryotic adaptive immune system (Shabbir et al. 2016), CRISPR/Cas enables accurate, rapid, cost‐effective, and efficient genome modifications by introducing targeted DNA insertions, deletions, or substitutions. This system is now widely applied in microbes, plants, and animals to generate desired phenotypes. In agriculture, CRISPR‐edited crops provide solutions to the challenges of increasing global food demand, limited arable land, and the slow genetic gains of conventional breeding. The technology has been successfully applied in over 20 major crops, including wheat, rice, maize, soybean, tomato, potato, cotton, and oilseeds, to improve yield, stress tolerance, and nutritional quality (Singh and Bokolia 2021; Kato‐Nitta et al. 2019).

CRISPR enables targeted genetic modification by deactivating or removing specific DNA sequences without introducing foreign DNA, resulting in non‐transgenic organisms. The edited site may be repaired using DNA from the same species (cisgenesis), a closely related species (intragenesis), or a sexually incompatible species (transgenesis). While transgenesis is generally classified as a GMO, the regulatory status of cisgenic and intragenic organisms remains debated and varies across countries.

## 8 Conclusion

This review demonstrates that the true power of CRISPR/Cas9 in cereal improvement lies not in the technology itself, but in its capacity to precisely reprogram enzymatic activities and metabolic pathways underlying complex agronomic traits. By targeting enzymes and regulatory nodes controlling hormone balance, carbon and nitrogen metabolism, photosynthesis, and defense responses, genome editing enables predictable modulation of yield, stress resilience, and grain quality.

Unlike conventional transgenic approaches, CRISPR/Cas‐based editing allows targeted reprogramming of key biological systems such as hormone signaling, photosynthesis, stress perception, and immune responses, thereby delivering predictable, heritable, and regulation‐friendly trait improvements in cereal crops. Yield and productivity are emergent properties of interconnected metabolic networks rather than outcomes of single‐gene action. CRISPR‐mediated trait improvement contributes to yield enhancement only when targeted edits reconfigure host metabolism, including carbon assimilation, hormone biosynthesis and turnover, nitrogen utilization, photosynthetic efficiency, and source–sink partitioning.

Edits in genes regulating cytokinin and gibberellin metabolism, starch biosynthesis enzymes, photosynthetic regulators, and stress‐signaling components alter enzymatic fluxes and physiological balances that collectively determine grain number, grain weight, and stress resilience. Consequently, future crop improvement strategies must prioritize pathway‐level and multiplex genome editing approaches that integrate metabolic, regulatory, and developmental controls rather than relying on isolated gene modifications.

Advances in transgene‐free editing, novel Cas variants, base and prime editing, and the integration of omics and artificial intelligence driven approaches will further expand the precision and scope of cereal genome engineering. However, translating laboratory success into field‐ready cultivars will require careful navigation of regulatory frameworks, intellectual property considerations, and freedom‐to‐operate challenges. Equally important are societal acceptance, ethical considerations, and farmer‐centric adoption strategies. While gene‐edited crops may benefit from relatively less restrictive regulatory oversight than transgenic organisms in many regions, international policy harmonization will be essential for widespread commercialization.

Ultimately, the impact of CRISPR‐based genome editing will be realized when pathway‐informed trait prioritization is strategically aligned with breeding objectives, agroecological requirements, and market demands. By integrating systems‐level genome editing with conventional and modern breeding approaches, it will be possible to rapidly develop resilient, high‐yielding, and consumer‐preferred cereal varieties, thereby contributing to global food and nutritional security under increasingly variable climatic conditions.

## Funding

The authors have nothing to report.

## Disclosure

The authors have nothing to report.

## Conflicts of Interest

The authors declare no conflicts of interest.

## Data Availability

The authors have nothing to report.
